# Fasting-mimicking diet enhances EGFR-TKI efficacy in oral cancer through dual mechanisms: direct cancer cell sensitization and tumor-associated macrophage crosstalk

**DOI:** 10.3389/fphar.2025.1641024

**Published:** 2025-07-30

**Authors:** Lei Wang, Yu-jie Wang, Rong Wang, Fu-lian Gong, Ji-Yuan Chen, Ya-ting Yu, Ze-rong Qiu, Yong-fang Yuan

**Affiliations:** ^1^Department of Pharmacy, Shanghai 9th People’s Hospital, Shanghai Jiao Tong University School of Medicine, Shanghai, China; ^2^National Children’s Medical Center, Shanghai Children’s Medical Center, School of Medicine, Shanghai Jiao Tong University, Shanghai, China

**Keywords:** fasting mimicking diets, EGFR-TKI, tumor-associated macrophages, oral cancer, CCL2, stat3

## Abstract

**Background:**

Emerging evidence suggests the fasting-mimicking diet (FMD) offers a promising alternative to traditional calorie restriction and intermittent fasting, mitigating associated adverse effects including cachexia. Clinical trials have demonstrated the safety and efficacy of FMD, highlighting its considerable potential for translational applications. Future research should focus on assessing with molecularly targeted therapies to enhance therapeutic outcomes. The present study investigates the efficacy of FMD combined with EGFR-TKI therapy in oral cancer.

**Methods:**

M2-polarized macrophages derived from THP-1 cells were used to model TAMs. 2D and 3D oral cancer cell cultures (Cal-27 and OECM-1) were treated with gefitinib under standard or FMD-conditioned media. TAMs recruitment and interaction with tumor spheroids were assessed via co-culture and Transwell assays. Cal-27 xenograft mouse model was used to evaluate *in vivo* effects of FMD and gefitinib. Gene expression and signaling pathways were analyzed through bioinformatics, ELISA, RT-PCR, Western blot, and immunohistochemistry.

**Results:**

FMD enhanced the anti-proliferative effect of gefitinib *in vitro* in both 2D and 3D oral cancer models directly. Bioinformatics and 3D models identified CCL2 as a gefitinib-induced chemokine reversed by FMD, which suppressed CCL2-mediated TAMs recruitment and tumor spheroid growth. *In vivo*, combined FMD and gefitinib treatment significantly reduced tumor volume, Ki-67+ proliferating cells, and M2-like TAMs density, accompanied by decreased serum CCL2 levels. Mechanistically, FMD inhibited gefitinib-induced STAT3 phosphorylation, leading to reduced CCL2 expression. Pharmacological modulation of STAT3 confirmed its role in regulating CCL2 secretion.

**Conclusion:**

In this study, we confirmed that fasting-mimicking diets not only directly enhances the sensitivity of oral cancer cells to gefitinib but also indirectly improves efficacy by attenuating CCL2-mediated TAMs recruitment under the gefitinib treatment environment. This study may provide a drug combination strategy and theoretical basis for the treatment of oral cancer, as well as scientific evidence for the clinical application of fasting-mimicking diets.

## Graphical Abstract

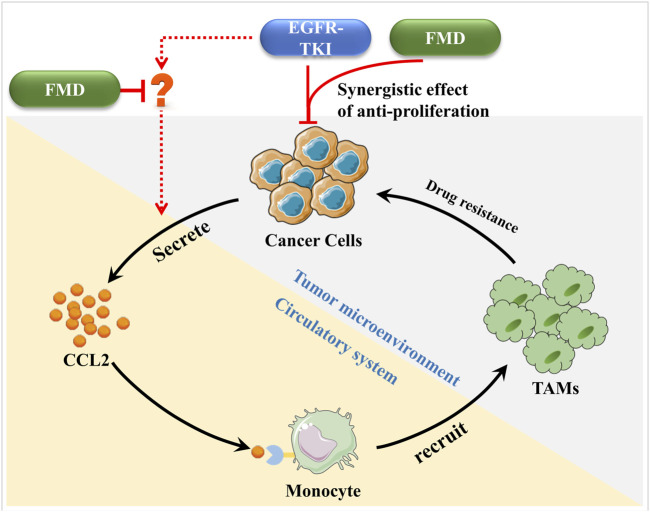

## 1 Introduction

Energy restriction such as calorie restriction and intermittent fasting (IF) demonstrate antitumor activity through metabolic reprogramming and treatment sensitization, yet their clinical application is hindered by cachexia-related risks (Martínez-Garay and Djouder, 2023). The fasting-mimicking diet (FMD)—characterized by cyclical low protein/sugar and high healthy fat intake—reduces cachexia risk without compromising therapeutic efficacy or patient compliance. Clinical studies have confirmed the safety and efficacy of FMD in cancer patients, highlighting its potential to synergize with chemotherapy and immunotherapy (Vernieri et al., 2022; de Groot et al., 2020; Salvadori et al., 2021; Cortellino et al., 2022; Ligorio et al., 2025; Nan et al., 2025). Mechanistic research indicates that FMD primarily remodeling the tumor microenvironment, promoting anti-tumor immunity (Vernieri et al., 2022; Cortellino et al., 2022; Zhong et al., 2023). Future research should focus on enhancing the combination of FMD with molecularly targeted therapies to improve treatment efficacy.

Our previous research also has provided evidence that FMD can attenuate the accumulation and pro-tumor function of tumor-associated macrophages (TAMs) in hypoxic tumor regions, suggesting its potential to improve the efficacy of anti-angiogenic treatments (Wang et al., 2023). Emerging research indicates that TAMs can diminish the efficacy of EGFR-targeted therapies through various mechanisms, including the secretion of exosomes and specific chemokines, modulation of intracellular signaling pathways, and promotion of EGFR degradation via increased oxidative stress (Chen et al., 2023; Xiao et al., 2020; Yuan et al., 2022; Yin et al., 2019).

In this study, we aim to assess the effects of FMD on TAMs within the gefitinib-treated microenvironment, with the goal of improving the anti-cancer efficacy of gefitinib in oral cancer. In this study, we aim to systematically evaluate the effects of FMD on TAMs within the gefitinib-treated microenvironment in oral cancer. We will focus on the STAT3/CCL2-TAMs axis, which has been implicated in the drug resistance mechanisms of oral squamous cell carcinoma (OSCC). By conducting a comprehensive analysis of existing literature and performing our own experimental studies, we seek to provide insights into the potential therapeutic synergy between FMD and gefitinib, with the ultimate goal of enhancing the therapeutic efficacy of EGFR-targeted treatments in oral cancer.

## 2 Materials and methods

### 2.1 Polarization of TAMs

In this study, we chose the M2 macrophage as the *in vitro* study object of TAMs. The steps of polarization are as follows: human monocytic cell line THP-1 (obtained from Type Culture Collection of the Chinese Academy of Sciences, Shanghai, China, passage number: 5–25) was stimulated with 50 mM phorbol 12-myristate 13-acetate (PMA, Beyotime, Haimen, China) for 24 h, and then differentiated into classical M2 macrophages by stimulation with 20 ng/mL interleukin-4 (IL-4) and interleukin-13 (IL-13, PeproTech, NJ, USA) for 72 h.

### 2.2 Establishment of 3D tumor spheroid model

First, prepare the non-adherent coating culture plate: add agarose powder to an appropriate amount of cell culture medium, and heat in a water bath at 80°C for 30 min until fully dissolved. After dissolution, quickly add the agarose solution to the 96-well plate at 60 μL per well, and allow the agarose to solidify for approximately 30 min. Subsequently, add the cell suspension pre-mixed with Matrigel matrix gel into the agarose-coated 96-well plate, and centrifuge at 4°C, 1,000 × g for 10 min. After 24 h of culture, observe the formation of spherical cell structures under a microscope.

### 2.3 3D spheroidr-monocyte coculture model

A cell suspension of tumor cells pre-stained with the green cell membrane dye DiO was added to a 96-well plate coated with agarose to prepare tumor microballs. After a 24-h incubation, monocytes (THP-1) pre-stained with the orange-red cell membrane dye Dil were introduced at a ratio of 1:4. Following a 7-day co-culture period, the tumor microballs were transferred to a new 96-well plate. After washing three times with PBS, fluorescence microscopy was performed for observation.

### 2.4 Cell counting Kit-8 (CCK-8) assay

For 2D cultures, 5 × 10^3^cells/well were seeded in 96-well plates. After 24 h attachment, cells were treated with gefitinib in either standard or FMD medium. CCK-8 reagent was added at 24 and 48 h. Absorbance at 450 nm was measured after 2 h incubation using a microplate reader. For 3D spheroids, Spheroids were washed with PBS (3×), dissociated into single cells. Cell viability was measured via CCK-8 after 4 h incubation.

### 2.5 Transwell Co-Culture model

THP-1 cells are seeded in the upper chamber of the Transwell and treated with PMA, IL-4, and IL-13 to differentiate THP-1 into M2-type TAMs. Next, tumor spheroids cultured for 7 days are transferred to the lower chamber of the Transwell and co-cultured with TAMs for 48 h. At the end of the experiment, any remaining cells on the surface of the upper chamber of the Transwell are removed using a cotton swab. The cells are then fixed with cold methanol-acetic acid, stained with crystal violet, and counted.

### 2.6 Fasting-mimicking diet medium

The *in vitro* fasting-mimicking diet medium was prepared following established protocols (Qian et al., 2022; Huang et al., 2023). The Fasting-mimicking diet medium consists of glucose-free medium supplemented with 0.5 g/L glucose and 5% FBS.

### 2.7 Fasting-mimicking diet

Before starting the study, the mice were fed the standard AIN-93G diet for 2 weeks, and their daily energy intake was monitored twice a week to establish a baseline. The FMD cycles consisted of a 'Day 1′and 'Day 2–3′ diet each week, with 10% animal-based protein and 90% fat. These diets provided 50% and 10% of the total energy intake from the baseline feeding, with a return to the normal diet at all other times.

### 2.8 Subcutaneous xenografted nude mice models of human oral cancer

BALB/c nude mice (5 weeks old) were purchased from Vital River Laboratories (Beijing, China). Cal-27 cells (1 × 10^7^/100 µL/each mouse) were injected into the armpits of each mouse. When the tumor volume reached 100–200 mm^3^, mice with excessively large or small tumors were excluded. The remaining mice were then randomly divided into treatment groups (n = 5): control; FMD; gefitinib (75 mg/kg/day, oral gavage (i.g.), MedChemExpress, NJ, USA); FMD + gefitinib (75 mg/kg/day, i.g.).

### 2.9 Gene set variation analysis (GSVA)

GSVA was conducted using the R package “GSVA” (version 1.52.5) implemented in R version 4.3.0 to assess the correlation of genes with specific gene sets. The gene sets used for this analysis were obtained from the Gene Set Enrichment Analysis (GSEA) database (https://www.gsea-msigdb.org).

### 2.10 Statistical analysis

Quantitative data were presented as mean ± SD, and group comparisons were made using one-way analysis of variance (ANOVA) with Tukey’s *post hoc* test. The statistical analyses were conducted using SPSS/Win 13.0 software (SPSS Inc., Chicago, USA).

## 3 Results

### 3.1 FMD directly enhances the anti-proliferative effect of gefitinib on oral cancer cells *in vitro*

**FIGURE 1:**
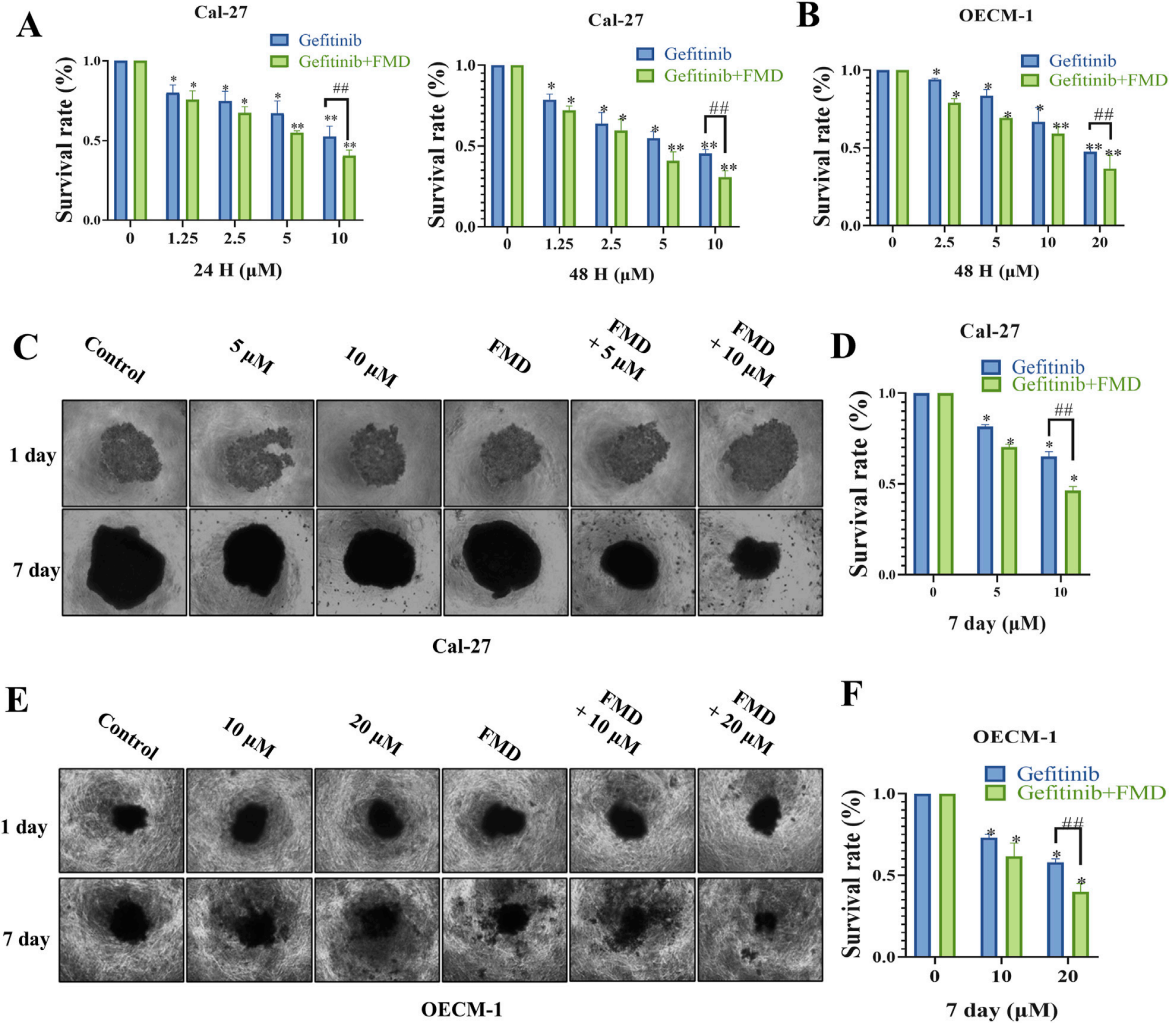
Fasting-mimicking diet (FMD) directly enhances the anti-proliferative effect of gefitinib on oral cancer cells *in vitro*. **(A,B)** Quantitative assessment of proliferation in Cal-27 cells and OECM-1 cells treated with gefitinib and FMD medium evaluated by CCK-8 assay. **(C,E)** 3D tumor spheroids Representative brightfield images of **(C)** Cal-27 cells, **(E)** OECM-1 cells, spheroids in monoculture. **(D,F)** Analysis of proliferation within 3D tumor spheroids constructed from Cal-27 or OECM-1 cells evaluated by CCK-8 assay. Data are presented as the means ± S.E.M. from three separate experiments. *P < 0.05 v.s. Control group, ##P < 0.01 v.s. Gefitinib group.

We investigated whether FMD medium enhances the effects of the EGFR inhibitor gefitinib on oral cancer in both 2D and 3D cell spheroid models. The results of the CCK-8 assay revealed that treatment with gefitinib in the 2D cell model led to a decrease in cell viability of Cal-27 cells in a dose-dependent and time-dependent manner (Figure 1A). Notably, with the survival rate of the combination group was normalized to the FMD-only group (not the control), gefitinib exhibited a markedly improved anti-tumor effect in the FMD-conditioned medium environment. Furthermore, similar observations were made in OECM-1 cells, where the combination of gefitinib and FMD demonstrated a synergistic effect (Figure 1B).

To further validate these findings, we constructed 3D tumor spheroids from both Cal-27 and OECM-1 cells and evaluated their proliferative capacity by CCK-8 assay. Consistent with the results from the 2D cell culture experiments, the combination of gefitinib and FMD significantly inhibited the growth of 3D tumor spheroids compared to either treatment alone (Figures 1C–F). These findings suggest that FMD acts synergistically with gefitinib to suppress the growth of oral cancer cells *in vitro*.

### 3.2 FMD inhibits gefitinib-induced CCL2 expression and secretion *in vitro*

**FIGURE 2:**
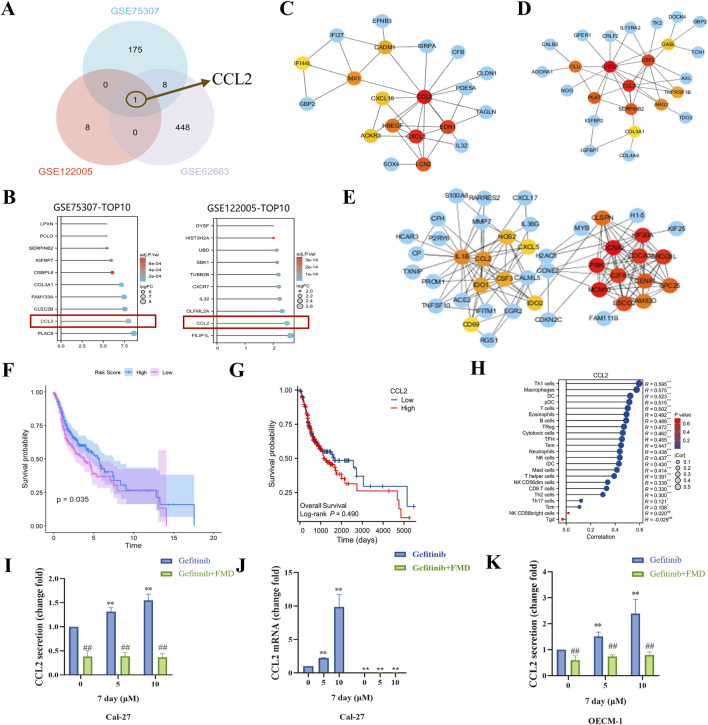
FMD inhibits gefitinib-induced CCL2 expression and secretion *in vitro*. **(A)** Venn diagram was used to analyze the intersection of elevated mRNA in GSE75307 and GSE122005 and decreased mRNA in GSE62663. **(B)** The top 10 genes were expressed in GSE122005 and GSE75307, respectively. C-E, protein-protein interaction (PPI) network analysis of elevated mRNA in GSE75307 **(C)** and GSE122005 **(D)** and decreased mRNA in GSE62663 **(E)**. **(F)** The correlation between Gefitinib induces elevated gensets (from GSE122005) and survival in oral cancer patients was investigated through analysis of the TCGA database. **(G)** The correlation between CCL2 expression and survival in oral cancer patients was investigated through analysis of the TCGA database. **(H)** Analysis of the TCGA database was conducted to explore the correlation between CCL2 and immune cell infiltration. **(I)** The CCL2 concentration in the supernatant of Cal-27 tumor spheroid culture was detected using ELISA. **(J)** Detection of CCL2 mRNA expression in Cal-27 tumor spheroids was performed through RT-PCR. **(K)** The CCL2 concentration in the supernatant of OECM-1 tumor spheroid culture was detected using ELISA. Data are presented as the means ± S.E.M. from three separate experiments. **P < 0.01 v.s. Control group of Gefitinib, ##P < 0.01 v.s. Control group of Gefitinib + FMD.

To investigate molecular mechanisms underlying the interaction between fasting-mimicking diet (FMD) and gefitinib, we conducted a multi-step bioinformatics analysis. First, cross-examination of Gene Expression Omnibus (GEO) datasets identified genes upregulated by gefitinib (GSE75307, GSE122005) and downregulated under nutrient deprivation (GSE62663). Venn analysis revealed CCL2 as the only overlapping candidate (Figure 2A), suggesting its potential role in mediating FMD-gefitinib crosstalk. This suggests that FMD interventions might reduce the gefitinib-induced upregulation of CCL2. Notably, CCL2 ranked among the top 10 upregulated genes in both gefitinib-treated datasets, indicating its consistent dysregulation under gefitinib treatment (Figure 2B). To further characterize CCL2’s functional relevance, protein-protein interaction (PPI) network analyses of gefitinib-upregulated genes from GSE75307 (Figure 2C) and GSE122005 (Figure 2D) consistently identified CCL2 as a central hub protein (Figure 2E). This network prominence implies that CCL2 may coordinate multiple downstream pathways activated by gefitinib.

Clinically, while GSVA scoring showed an inverse correlation between the gefitinib-induced gene set and poor prognosis in head and neck cancer (The Cancer Genome Atlas (TCGA) data, Figure 2F), CCL2 exhibited a distinct pattern: its elevated expression significantly associated with worse survival outcomes (Figure 2G) and enhanced macrophage infiltration (Figure 2H). This paradoxical observation suggests that although the broader gefitinib response signature may confer protective effects, CCL2 specifically could drive tumor-associated macrophage (TAMs) recruitment, potentially counteracting therapeutic efficacy by remodeling the tumor microenvironment.

To validate these computational predictions, we performed functional experiments in oral cancer cell models. LISA and RT-PCR assays demonstrated that gefitinib dose-dependently reduced CCL2 secretion in Cal-27 tumor spheroids (Figure 2I), while FMD significantly reversed gefitinib-induced CCL2 suppression at both protein (Figure 2I) and mRNA levels (Figure 2J). Similar trends were observed in OECM-1 cells (Figure 2K), confirming the inhibitory effect of FMD on gefitinib-mediated CCL2 modulation. These results mechanistically support the hypothesis that FMD may suppress gefitinib-induced TAMs recruitment by specifically targeting CCL2 production.

### 3.3 FMD disrupts CCL2-mediated TAMs recruitment to potentiate gefitinib efficacy *in vitro*

**FIGURE 3:**
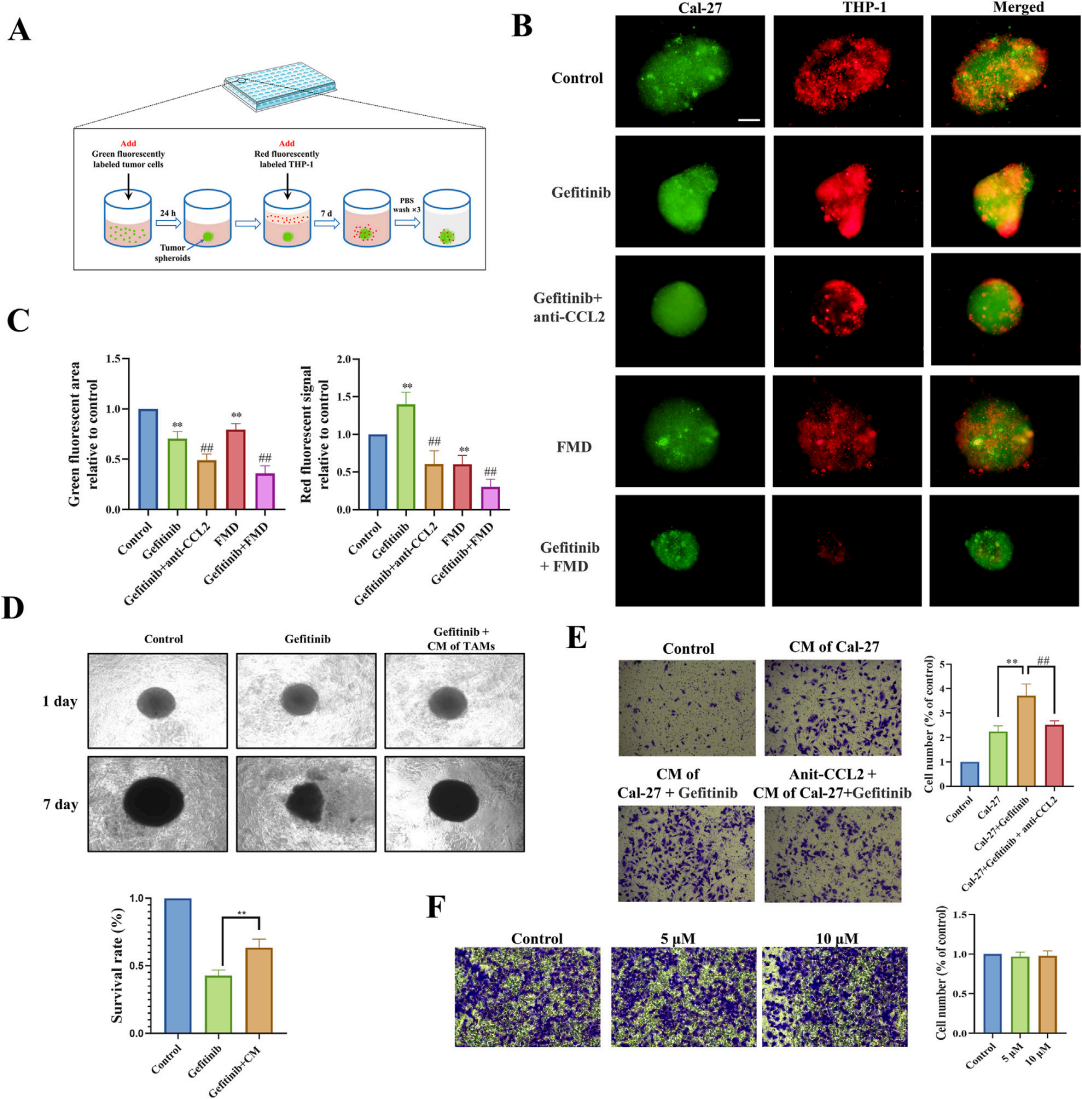
FMD disrupts CCL2-mediated TAMs recruitment to potentiate gefitinib efficacy *in vitro*. **(A)** Schematic diagram of 3D tumor spheroid-macrophage co-culture model. **(B,C)** Immunofluorescence and fluorescence statistics were used to observe the recruitment effect of Cal-27 tumor microspheres on macrophages (magnification ×10). **(D)** Microscopic observation and CCK-8 were used to detect the effect of TAMs-conditioned medium on the sensitivity of Cal-27 tumor spheroids to gefitinib. **(E)** Transwell assay was used to observe the effect of Cal-27 cell-conditioned medium on TAMs migration (magnification ×40). **(F)** Transwell was used to detect the effect of gefitinib on the migration of TAMs (48 h). **P < 0.01 v.s. Control group, ##P < 0.01 v.s. Gefitinib group.

To functionally validate the CCL2-TAMs recruitment axis identified above, we developed a 3D Cal-27 tumor spheroid-macrophage co-culture system (Figure 3A). Quantitative fluorescence imaging demonstrated that gefitinib treatment significantly enhanced TAMs infiltration into tumor spheroids compared to controls (Figures 3B,C). Notably, both the neutralizing antibody targeting CCL2 and the FMD medium significantly inhibited gefitinib-induced macrophage recruitment and concurrently suppressed tumor spheroid growth. This functional interdependence between immune microenvironment remodeling and tumor growth kinetics suggests CCL2-mediated chemotaxis constitutes a critical resistance mechanism.

To dissect the mechanism underlying TAMs-mediated therapeutic resistance, we established a paracrine communication model using conditioned media exchange. Tumor spheroids exposed to TAMs-derived conditioned media exhibited significantly reduced gefitinib sensitivity (Figure 3D), indicating that TAMs-secreted factors confer drug resistance. Additionally, Transwell co-culture experiments showed that conditioned medium of gefitinib-treated Cal-27 significantly increased the recruitment of TAMs, while the CCL2 neutralizing antibody effectively countered this promotion (Figure 3E). Importantly, control experiments confirmed that gefitinib does not directly stimulate TAMs migration (Figure 3F).

Our integrated findings establish a feedforward resistance loop: Gefitinib treatment paradoxically enhances tumor cell CCL2 secretion, driving TAMs infiltration that subsequently secretes protective factors to diminish drug efficacy. FMD intervention disrupts this pathogenic cycle through dual mechanisms - directly impairing tumor cell CCL2 production and indirectly modulating TAMs effector functions - thereby resensitizing malignancies to EGFR-targeted therapy.

### 3.4 FMD synergizes with gefitinib to suppress oral tumorigenesis through CCL2-TAMs axis modulation *in vivo*

**FIGURE 4:**
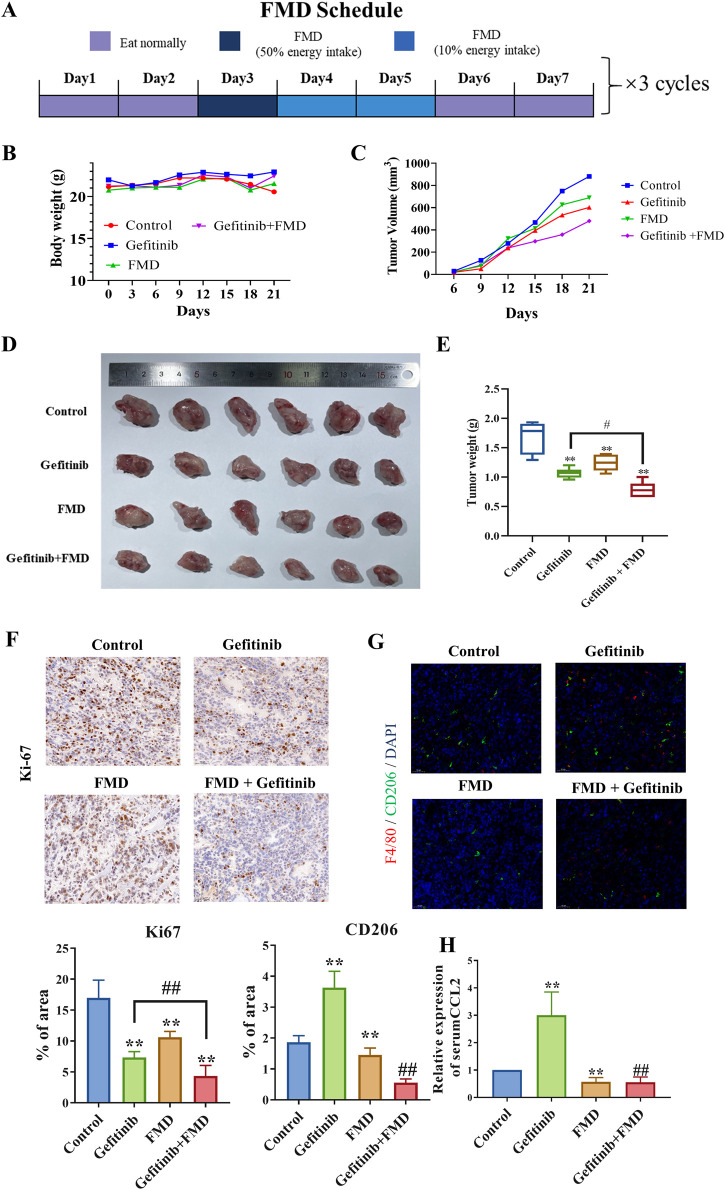
The combination of FMD and gefitinib synergistically suppressed mice oral tumor growth. Mice were then randomly divided into treatment groups (n = 5): control; FMD; gefitinib (75 mg/kg/day, oral gavage (i.g.)); FMD + gefitinib (75 mg/kg/day, i.g.). **(A)** Schematic diagram of the FMD diet plan. **(B)** Mice body weights recorded every 3 days. **(C)** Mouse tumor volumes recorded every 3 days. **(D)** Representative tumor tissue from each group. **(E)** Tumor weights in each group. **(F)** Ki-67 expression in tumor tissues was detected by immunohistochemistry (magnification ×20). **(G)** F4/80 (red) and CD206 (green) expression in tumor tissues was detected by immunofluorescence (magnification ×20). **(H)** Serum CCL8 level in mice is detected by ELISA. n = 6 **P < 0.01 v.s. Control group, ##P < 0.01 v.s. Gefitinib group.

To evaluate the translational potential of our findings, we employed a Cal-27 xenograft model, selected for its sensitivity to EGFR signaling (Hu et al., 2024), to recapitulate disease progression. Treatment with FMD for a total of three cycles resulted in a notable reduction in tumor volume compared to the control group. Additionally, mice treated with a combination of FMD and gefitinib (75 mg/kg/day) showed a further decrease in tumor volume compared to those treated with gefitinib alone (Figures 4C–E).

Histopathological analysis revealed that combined therapy induced tumor stromal remodeling, evidenced by 74% reduction in Ki-67+ proliferating tumor cells (Figure 4F) and 65% decrease in CD163+ M2-like TAMs density (Figure 4G). Notably, ELISA results further demonstrated that FMD treatment significantly lowered the serum CCL2 levels in the gefitinib-treated mice (Figure 4H).

### 3.5 FMD attenuates gefitinib-induced CCL2 expression through STAT3 phosphorylation inhibition

**FIGURE 5:**
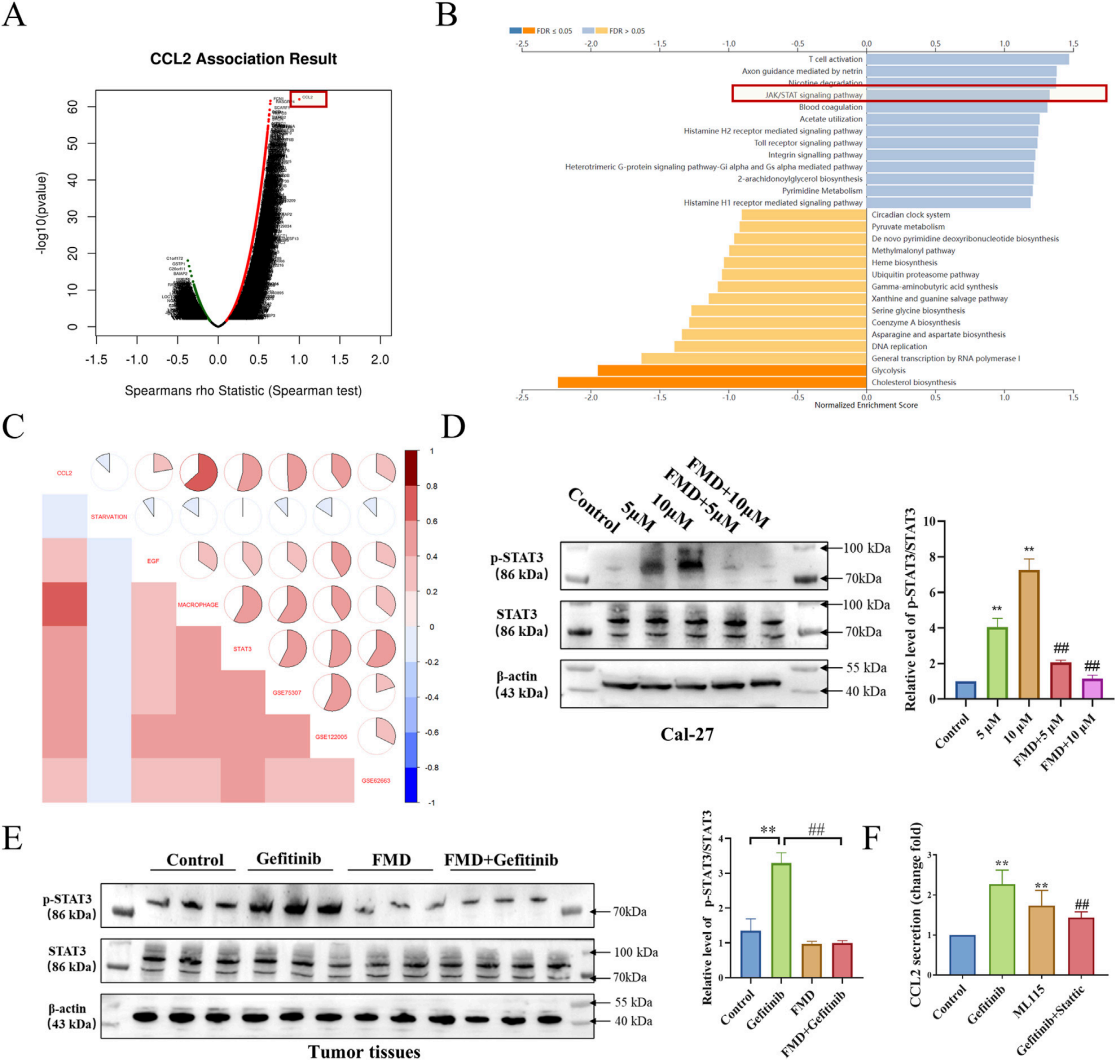
FMD attenuates gefitinib-Induced CCL2 expression through STAT3 phosphorylation inhibition. **(A)** Analysis of positively correlated genes of CCL2 in TCGA-HNSC using the LinkedOmics database. **(B)** Analysis of signaling pathways related to positively correlated genes (Top 500) of CCL2 using the LinkedOmics database. **(C)** GSVA analysis of correlation between CCL2, glucose starvation (Gene set from GESA M15497), EGF (MM1386), macrophage (M39708), STAT3 (M1163), elevated mRNA in GSE75307, GSE122005 and decreased mRNA in GSE62663 in samples from TCGA-HNSC. **(D)** Western blot analysis was performed to evaluate the effects of 5 μM and 10 μM gefitinib alone and in combination with FMD for 7 days on the phosphorylation of STAT3 in Cal-27 tumor spheroids. **(E)** Western blot analysis was performed to measure the level of STAT3 phosphorylation in tumor tissues. **(F)** CCL2 secretion from Cal-27 cell treated with Stattic (10 μM), ML115 (10 μM) for 24 h is determined by ELISA (*P < 0.05, v.s respective TAMs group). Data are presented as the means ± S.E.M. from three separate experiments. **P < 0.01 v.s. Control group, ##P < 0.01 v.s. Gefitinib.

To elucidate the molecular mechanisms underlying FMD-mediated suppression of gefitinib-induced CCL2 expression, we employed a multi-modal analytical approach. Gene Set Enrichment Analysis (GSEA) of TCGA-HNSC patient data indicated that CCL2-related gene pathways include the STAT signaling pathway (Figures 5A,B), prompting subsequent prioritization of STAT3 over c-Jun for mechanistic investigation. Furthermore, gene set variation analysis (GSVA) demonstrated a positive correlation between CCL2 and gene sets associated with EGF, macrophages, gefitinib, and genes induced by gefitinib from datasets GSE75307 and GSE122005. Conversely, a negative correlation was observed with gene sets related to glucose starvation in head and neck squamous cell carcinoma samples (Figure 5C). These multi-omics findings collectively suggest STAT3 phosphorylation as a critical mediator of gefitinib-induced CCL2 upregulation.

To validate the above hypothesis, we observed the effects of gefitinib, FMD, and their combination on the phosphorylation of STAT3 in both oral cancer cell lines Cal-27 and mouse tumor tissues. Western blot results (Figures 5D,E) showed that at the cellular level and in mouse tumor tissues, gefitinib can induce STAT3 phosphorylation, while FMD inhibits gefitinib-induced STAT3 phosphorylation. Additionally, in the *in vitro* cell model, ELISA results indicated that the gefitinib-induced secretion of CCL2 was diminished by the STAT3 inhibitor Stattic, while the STAT3 agonist ML115 enhanced CCL2 secretion (Figure 5F). These dose-responsive, reciprocal effects confirm STAT3 activation status as a critical determinant of CCL2 expression levels in the combined effects of FMD and gefitinib.

## 4 Discussion

Energy restriction has attracted attention for its ability to enhance the efficacy of various anti-cancer therapies, including chemotherapy and immunotherapy (Pateras et al., 2023). Our study extends these findings, demonstrating that FMD enhances the sensitivity of oral squamous cell carcinoma cells to gefitinib, thereby underscoring the therapeutic potential of this dietary intervention.

Given the critical involvement of TAMs in cancer progression and response to EGFR-targeted therapies, we sought to assess the role of TAMs in combined effects of FMD and gefitinib. Numerous studies have shown that TAMs can compromise the effectiveness of EGFR-targeted treatments through various mechanisms, such as the secretion of exosomes and chemokine CCL15, modulation of cellular signaling pathways in cancer cells, and promotion of EGFR degradation through increased reactive oxygen species (ROS) (Chen et al., 2023; Xiao et al., 2020; Yuan et al., 2022; Yin et al., 2019). Consistent with this established role in resistance, our data reveal that gefitinib treatment increases the infiltration of TAMs, which subsequently reduces tumor cell sensitivity to the drug. This observation is consistent with current literature that highlights TAMs modulation as a critical factor target for enhancing gefitinib efficacy.

Energy restriction, a hallmark of dietary interventions such as FMD, is known to modulate macrophage function across various pathological conditions. Specifically, energy restriction can inhibit the migration of monocytes (macrophage precursors) from the bone marrow to tissues in both healthy humans and mice by suppressing the production of CCL2, a key chemoattractant for TAMs recruitment (Jordan et al., 2019; Feng et al., 2025). Notably, previous reports have indicated that gefitinib upregulates CCL2 expression (Xiao et al., 2020; Yamaki et al., 2010; Jia et al., 2019), suggesting its potential role in promoting TAMs accumulation. In this study, we found that FMD can inhibit the gefitinib-induced CCL2 expression. Moreover, the reduction in CCL2 due to FMD also led to decreased TAMs recruitment, enhancing gefitinib’s therapeutic efficacy. These results suggest that FMD may effectively inhibit TAMs recruitment, supporting the development of combination strategies.

The complex treatment landscape of tumors necessitates a multi-faceted approach toward therapeutic intervention. The synergism between FMD and gefitinib may extend beyond the mechanisms outlined here, warranting further investigation into aspects such as anti-angiogenesis. FMD exerts broader immunomodulatory effects beyond macrophages; for instance, emerging evidence suggests FMD may enhance anti-tumor immunity by modulating T-cell activity and other immune populations (Vernieri et al., 2022). Thus, further studies are warranted to elucidate whether FMD’s synergy with EGFR-targeted therapy involves complementary effects on additional immune cells. Such aspects include anti-angiogenesis. Given that gefitinib, TAMs, and CCL2 are all interconnected with tumor vascularization processes (Singh et al., 2023; Dallavalasa et al., 2021; O'Connor and Heikenwalder, 2021), it is plausible that FMD influences gefitinib’s anti-angiogenic effect. Moreover, the side effects associated with gefitinib, which include dermal toxicity, diarrhea, and pulmonary complications, are intimately linked to macrophage activity (Wan et al., 2020; Cao et al., 2024; Du et al., 2021; Wang et al., 2024). Consequently, the combination regimen of FMD and EGFR targeting may also attenuate these toxicities.

In this study, we explored the potential of FMD as an adjunctive strategy to enhance gefitinib efficacy in treating oral tumor in mice and 3D cell models. Our results reveal that FMD significantly increases the sensitivity of oral tumor cells to gefitinib. Specifically, we found that gefitinib treatment leads to an increase in tumor-associated macrophages, which reduces the drug’s effectiveness. Importantly, FMD was shown to suppress gefitinib-induced CCL2 expression by inhibiting the phosphorylation of STAT3. This inhibition of CCL2 expression by FMD reduces TAMs recruitment, thereby improving the therapeutic response to gefitinib. Overall, FMD offers a promising approach to augment gefitinib therapy and improve clinical outcomes in oral cancer treatment.

## Data Availability

Publicly available datasets were analyzed in this study. This data can be found here: The RNA-seq data (GSE75307, GSE122005, GSE62663) are accessible via the Gene Expression Omnibus (https://www.ncbi.nlm.nih.gov/geo/). Normalized data from the TCGA-HNSC cohort can be obtained through UCSC Xena (http://xena.ucsc.edu/).

## References

[B1] Cao J. Chen S. Wang J. Fan X. Liu S. Li X. (2024). Transcription factor PRRX1-activated ANXA6 facilitates EGFR-PKCα complex formation and enhances cisplatin sensitivity in bladder cancer. Life Sci. 359, 123228. 10.1016/j.lfs.2024.123228 39528080

[B2] Chen M. T. Li B. Z. Zhang E. P. Zheng Q. (2023). Potential roles of tumor microenvironment in gefitinib-resistant non-small cell lung cancer: a narrative review. Med. Baltim. 102, e35086. 10.1097/md.0000000000035086 PMC10553124 37800802

[B3] Cortellino S. Raveane A. Chiodoni C. Delfanti G. Pisati F. Spagnolo V. (2022). Fasting renders immunotherapy effective against low-immunogenic breast cancer while reducing side effects. Cell Rep. 40, 111256. 10.1016/j.celrep.2022.111256 36001966

[B4] Dallavalasa S. Beeraka N. M. Basavaraju C. G. Tulimilli S. V. Sadhu S. P. Rajesh K. (2021). The role of tumor associated macrophages (TAMs) in cancer progression, chemoresistance, angiogenesis and metastasis - current status. Curr. Med. Chem. 28, 8203–8236. 10.2174/0929867328666210720143721 34303328

[B5] de Groot S. Lugtenberg R. T. Cohen D. Welters M. J. P. Ehsan I. Vreeswijk M. P. G. (2020). Fasting mimicking diet as an adjunct to neoadjuvant chemotherapy for breast cancer in the multicentre randomized phase 2 DIRECT trial. Nat. Commun. 11, 3083. 10.1038/s41467-020-16138-3 32576828 PMC7311547

[B6] Du J. Li G. Jiang L. Zhang X. Xu Z. Yan H. (2021). Crosstalk between alveolar macrophages and alveolar epithelial cells/fibroblasts contributes to the pulmonary toxicity of gefitinib. Toxicol. Lett. 338, 1–9. 10.1016/j.toxlet.2020.11.011 33248157

[B7] Feng Q. Li C. Z. Zou Y. H. Wang X. Y. Yang X. Zhang R. (2025). IL6/CCL2 from M2-polarized microglia promotes breast cancer brain metastasis and the reversal effect of β-elemene. Front. Pharmacol. 16, 1547333. 10.3389/fphar.2025.1547333 40444044 PMC12119470

[B8] Hu S. Li S. Xu Y. Huang X. Mai Z. Chen Y. (2024). The antitumor effects of herbal medicine Triphala on oral cancer by inactivating PI3K/Akt signaling pathway: based on the network pharmacology, molecular docking, *in vitro* and *in vivo* experimental validation. Phytomedicine 128, 155488. 10.1016/j.phymed.2024.155488 38493718

[B9] Huang W. Li X. Song H. Yin Y. Wang H. (2023). Verification of fasting-mimicking diet to assist monotherapy of human cancer-bearing models. Biochem. Pharmacol. 215, 115699. 10.1016/j.bcp.2023.115699 37482198

[B10] Jia Y. Li X. Jiang T. Zhao S. Zhao C. Zhang L. (2019). EGFR-targeted therapy alters the tumor microenvironment in EGFR-driven lung tumors: implications for combination therapies. Int. J. Cancer 145, 1432–1444. 10.1002/ijc.32191 30784054

[B11] Jordan S. Tung N. Casanova-Acebes M. Chang C. Cantoni C. Zhang D. (2019). Dietary intake regulates the circulating inflammatory monocyte pool. Cell 178, 1102–1114.e17. 10.1016/j.cell.2019.07.050 31442403 PMC7357241

[B12] Ligorio F. Vingiani A. Torelli T. Sposetti C. Drufuca L. Iannelli F. (2025). Early downmodulation of tumor glycolysis predicts response to fasting-mimicking diet in triple-negative breast cancer patients. Cell metab. 37, 330–344.e7. 10.1016/j.cmet.2024.11.004 39694040

[B13] Martínez-Garay C. Djouder N. (2023). Dietary interventions and precision nutrition in cancer therapy. Trends Mol. Med. 29, 489–511. 10.1016/j.molmed.2023.04.004 37263858

[B14] Nan K. Zhong Z. Yue Y. Shen Y. Zhang H. Wang Z. (2025). Fasting-mimicking diet-enriched Bifidobacterium pseudolongum suppresses colorectal cancer by inducing memory CD8(+) T cells. Gut 74, 775–786. 10.1136/gutjnl-2024-333020 39870395

[B15] O'Connor T. Heikenwalder M. (2021). CCL2 in the tumor microenvironment. Adv. Exp. Med. Biol. 1302, 1–14. 10.1007/978-3-030-62658-7_1 34286437

[B16] Pateras I. S. Williams C. Gianniou D. D. Margetis A. T. Avgeris M. Rousakis P. (2023). Short term starvation potentiates the efficacy of chemotherapy in triple negative breast cancer via metabolic reprogramming. J. Transl. Med. 21, 169. 10.1186/s12967-023-03935-9 36869333 PMC9983166

[B17] Qian R. Cao G. Su W. Zhang J. Jiang Y. Song H. (2022). Enhanced sensitivity of tumor cells to autophagy inhibitors using fasting-mimicking diet and targeted lysosomal delivery nanoplatform. Nano Lett. 22, 9154–9162. 10.1021/acs.nanolett.2c03890 36342406

[B18] Salvadori G. Zanardi F. Iannelli F. Lobefaro R. Vernieri C. Longo V. D. (2021). Fasting-mimicking diet blocks triple-negative breast cancer and cancer stem cell escape. Cell metab. 33, 2247–2259.e6. 10.1016/j.cmet.2021.10.008 34731655 PMC8769166

[B19] Singh S. Sadhukhan S. Sonawane A. (2023). 20 years since the approval of first EGFR-TKI, gefitinib: insight and foresight. Biochim. Biophys. Acta Rev. Cancer 1878, 188967. 10.1016/j.bbcan.2023.188967 37657684

[B20] Vernieri C. Fucà G. Ligorio F. Huber V. Vingiani A. Iannelli F. (2022). Fasting-mimicking diet is safe and reshapes metabolism and antitumor immunity in patients with cancer. Cancer Discov. 12, 90–107. 10.1158/2159-8290.cd-21-0030 34789537 PMC9762338

[B21] Wan L. Wang Y. Tang Y. Tan Y. He F. Zhang Y. (2020). Gefitinib-induced cutaneous toxicities in Brown Norway rats are associated with macrophage infiltration. Inflammation 43, 2137–2146. 10.1007/s10753-020-01281-2 33025329

[B22] Wang L. Wang Y. J. Wang R. Gong F. L. Shi Y. H. Li S. N. (2023). Fasting mimicking diet inhibits tumor-associated macrophage survival and pro-tumor function in hypoxia: implications for combination therapy with anti-angiogenic agent. J. Transl. Med. 21, 754. 10.1186/s12967-023-04577-7 37884960 PMC10601181

[B23] Wang X. Cheng X. Liu H. Mu X. Zheng H. (2024). Food nutrition and toxicology targeting on specific organs in the era of single-cell sequencing, Food Sci. Hum. Wellness. 13 75–89. 10.26599/FSHW.2022.9250006

[B24] Xiao F. Liu N. Ma X. Qin J. Liu Y. Wang X. (2020). “M2 macrophages reduce the effect of gefitinib by activating AKT/mTOR in gefitinib-resistant cell lines HCC827/GR,”, 11. Thorac Cancer, 3289–3298. 10.1111/1759-7714.13670 PMC7606002 32956565

[B25] Yamaki M. Sugiura K. Muro Y. Shimoyama Y. Tomita Y. (2010). Epidermal growth factor receptor tyrosine kinase inhibitors induce CCL2 and CCL5 via reduction in IL-1R2 in keratinocytes. Exp. Dermatol 19, 730–735. 10.1111/j.1600-0625.2010.01108.x 20590818

[B26] Yin X. Han S. Song C. Zou H. Wei Z. Xu W. (2019). Metformin enhances gefitinib efficacy by interfering with interactions between tumor-associated macrophages and head and neck squamous cell carcinoma cells. Cell Oncol. (Dordr) 42, 459–475. 10.1007/s13402-019-00446-y 31001733 PMC12994286

[B27] Yuan S. Chen W. Yang J. Zheng Y. Ye W. Xie H. (2022). Tumor-associated macrophage-derived exosomes promote EGFR-TKI resistance in non-small cell lung cancer by regulating the AKT, ERK1/2 and STAT3 signaling pathways. Oncol. Lett. 24, 356. 10.3892/ol.2022.13476 36168315 PMC9478622

[B28] Zhong Z. Zhang H. Nan K. Zhong J. Wu Q. Lu L. (2023). Fasting-mimicking diet drives antitumor immunity against colorectal cancer by reducing IgA-producing cells. Cancer Res. 83, 3529–3543. 10.1158/0008-5472.Can-23-0323 37602826 PMC10618736

